# Chemical signature of colorectal cancer: case–control study for profiling the breath print

**DOI:** 10.1002/bjs5.50354

**Published:** 2020-09-29

**Authors:** D. F. Altomare, A. Picciariello, M. T. Rotelli, M. De Fazio, A. Aresta, C. G. Zambonin, L. Vincenti, P. Trerotoli, N. De Vietro

**Affiliations:** ^1^ Surgical Unit ‘M. Rubino’, Department of Emergency and Organ Transplantation Bari Italy; ^2^ Statistical Unit, Department of Biomedical Sciences and Human Oncology Bari Italy; ^3^ Department of Chemistry University Aldo Moro of Bari Bari Italy; ^4^ Apulian Breath Analysis Centre (CeRBA) Istituto Di Ricovero e Cura a Carattere Scientifico (IRCCS) Istituto Tumori Giovanni Paolo II Bari Italy; ^5^ Surgical Unit Azienda Ospedaliero‐Universitaria Policlinico Bari Bari Italy

## Abstract

**Background:**

Effective screening for colorectal cancer can reduce mortality by early detection of tumours and colonic polyps. An altered pattern of volatile organic compounds (VOCs) in exhaled breath has been proposed as a potential non‐invasive diagnostic tool for detection of cancer. The aim of this study was to evaluate the reliability of breath‐testing for colorectal cancer screening and early diagnosis using an advanced breath sampler.

**Methods:**

The exhaled breath of patients with colorectal cancer and non‐cancer controls with negative findings on colonoscopy was collected using the ReCIVA® Breath Sampler. This portable device is able to capture the alveolar breath fraction without environmental contamination. VOCs were desorbed thermally and analysed by gas chromatography–mass spectrometry. The discriminatory ability of VOCs in detecting colorectal cancer was evaluated by receiver operating characteristic (ROC) curve analysis for each VOC, followed by cross‐validation by the leave‐one‐out method, and by applying stepwise logistic regression analysis.

**Results:**

The study included 83 patients with colorectal cancer and 90 non‐cancer controls. Fourteen VOCs were found to have significant discriminatory ability in detecting patients with colorectal cancer. The model with the diagnosis of cancer *versus* no cancer resulted in a statistically significant likelihood of discrimination of 173·45 (*P* < 0·001), with an area under the ROC curve of 0·979. Cross‐validation of the model resulted in a true predictive value for colorectal cancer of 93 per cent overall. Reliability of the breath analysis was maintained irrespective of cancer stage.

**Conclusion:**

This study demonstrated that analysis of exhaled VOCs can discriminate patients with colorectal cancer from those without. This finding may eventually lead to the creation of a smart online sensory device, capable of providing a binary answer (cancer/no cancer) and directing to further screening.

## Graphical abstract

Available screening methods for colorectal cancer have poor reliability and low patient compliance. Cancer cells produce altered metabolites, which are transported in the bloodstream and released in the alveoli; these volatile organic compounds (VOCs) can be detected in exhaled breath. This study used a new‐generation breath sampler, capable of selecting the alveolar fraction of the breath and preventing environmental contamination. A pattern of 14 VOCs was able to discriminate patients with colorectal cancer from healthy controls, with a true predictive value of 93 per cent, irrespective of cancer stage.

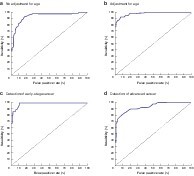

Breath test signature in colorectal cancer

## Abstract (es)

**Antecedentes:**

Un cribaje efectivo del cáncer colorrectal (*colorectal cáncer*, CRC) puede reducir la mortalidad mediante la detección precoz de cáncer/pólipos del colon. La identificación de un patrón de compuestos volátiles orgánicos (*volatile organic compounds*, VOCs) en el aire espirado se ha propuesto como un procedimiento potencial de diagnóstico no invasivo para la detección del cáncer. El objetivo de este estudio fue evaluar la factibilidad del test de la respiración para el cribaje del CRC y diagnóstico precoz empleando un equipo avanzado de muestreo del aliento.

**Métodos:**

Se recogieron muestras de aire espirado de 83 pacientes con CRC y de 90 controles sin cáncer con colonoscopia negativa empleando el ReCIVA Breath Sampler©. Este equipo portátil es capaz de capturar la fracción de aire alveolar espirada ausente de contaminación ambiental. Los VOCs fueron aislados térmicamente y analizados mediante cromatografía de gases acoplada a espectrometría de masas. La capacidad discriminatoria de los VOCs para detectar pacientes con CCR se evaluó mediante un análisis de la curva ROC para cada VOC seguida de validación cruzada mediante el método ir eliminando paso a paso cada uno de los VOCs en un modelo de regresión logística.

**Resultados:**

Se observó que 14 VOCs tenían habilidad discriminatoria significativa para la detección de pacientes con CRC. El modelo con el diagnóstico de cáncer *versus* no cáncer mostró una probabilidad estadísticamente significativa de 151,03 (*P* < 0,0001) con un área bajo la curva (*area under the curve*, AUC) de 0,963. En la validación cruzada del modelo se obtuvo un valor global predictivo verdadero para el CRC del 92,5%. La fiabilidad del análisis del aire espirado se mantuvo con independencia del estadio del cáncer.

**Conclusión:**

Este estudio ha demostrado que el análisis de los VOCs en el aire espirado puede discriminar pacientes con CRC de pacientes sin cáncer. Este hallazgo podría ser de ayuda para diceñar un dispositivo sensorial inteligente en línea, capaz de proporcionar una respuesta binaria (cáncer/NO cáncer) y asimismo contribuir a la indicación de una futura colonoscopia.

## Introduction

Colorectal cancer is amongst the four biggest cancer killers in the European Union^1^, and the second most frequent cause of death in Italy^2^. Given the encouraging data with selected endoscopic screening and early polypectomy with regard to cancer prevention and survival advantage^3^, there is an ongoing search within national health programmes for effective and reliable screening methods. Recently, breath‐testing has been pursued as an option, and a range of volatile organic compounds (VOCs) present in the exhaled breath of patients with cancer have been identified^4^. Several groups have reported specific VOCs for cancer diagnosis, not only for colorectal cancer^4^, ^5^, ^6^, ^7^, ^8^ but also for cancers of the lung^9^, ^10^ stomach^11^, ^12^ pancreas^13^ and breast^14^; each cancer type is associated with a unique breath print.

Currently, a wide variety of VOC candidates have been identified in colorectal cancer, probably reflecting the use of varying substrates and analytical platforms^15^. In 2013, the present authors' group published the first study^4^ outlining the potential of breath analysis for colorectal cancer screening, using a customized breath sampler and gas chromatography–mass spectrometry (GC‐MS) analysis. That study identified a pattern of 15 specific VOCs that had discriminatory capacity between patients with colorectal cancer and controls. Since then, other groups^6^, ^7^, ^16^ have independently published a variety of different VOC breath patterns in colorectal cancer, using similar methodologies.

The aim of the present case–control study was to evaluate whether the addition of a new advanced breath sampler could specifically capture only the alveolar breath fraction and exclude environmental contaminants effectively.

## Methods

The study was approved by the ethics committee of the Azienda Ospedaliero‐Universitaria Policlinico, Bari, Italy, and performed in compliance with the Declaration of Helsinki; it was registered at https://clinicaltrials.gov (identifier NCT04217083). All of the patients and non‐cancer controls recruited provided written informed consent before breath‐testing.

The study recruited patients of any sex or age undergoing curative surgery for histologically proven adenocarcinoma of the colon or rectum (of any clinical stage) and a comparative group of non‐cancer controls who had negative findings on colonoscopy performed within the previous 3 years. The control group was recruited from patients who had undergone colonoscopy for occult or overt rectal bleeding, or as part of diagnostic workup for chronic constipation. Patients excluded from analysis were those who had any history of another type of cancer, previous endoscopic removal of colonic polyps or a history of familial adenomatous polyposis or Lynch syndrome, and a history of active inflammatory disorder or liver disease. Patients with diverticulosis, chronic constipation or haemorrhoids were included in the analysis, as were those who were smokers or who had diabetes and/or hypertension.

A dedicated electronic database was established to analyse the data. To test the reliability of the pattern of VOCs in patients with colorectal cancer, patients were divided according to clinical cancer stage^17^ into two main staging subgroups: early (stage I–II) and advanced (stage III–IV) stage disease.

Exhaled breath was collected with the ReCIVA® Breath Sampler (Owlstone Medical, Cambridge, UK). The device was connected to a breath‐sampling kit (mask and sorbent tubes), ensuring reproducible collection of VOCs during real‐time monitoring of the patient's breathing. The exhaled breath of each patient was captured into four carbon tubes containing a mixed formulation of Tenax® and Carbograph® (Markes International, Llantrisant, UK) capable of retaining a range of carbon compounds (from C4 to C30). The apparatus comprised infrared carbon dioxide detection with pressure sensors, permitting the selection of different volumes and fractions of the exhaled breath. A mask manufactured from medical grade silicone, which included a high‐efficiency, low‐resistance bacterial filter, was fixed on to the device before each sampling. This was connected to a medical air canister via a plastic pressure reducer, set to 15 litres/min.

A USB cable connected the ReCIVA® breath sampler to a laptop installed with breath‐sampling software (Owlstone Medical), designed to ensure accurate monitoring of breathing air pressures (partial pressure of carbon dioxide). All subjects were fasted for at least 4 h before breath sampling. Sampling was always performed in the same room, aerated for 30 min before each procedure. Patients were instructed to keep the mask securely adhered to their face and to breathe normally the air released by the medical air canister. After a 60‐s ReCIVA® device washout with pure air (purity 99·99 per cent; SOL Group, Monza, Italy), patient breath was collected for 10 min under PC‐dedicated program control. At the completion of sampling the sorbent tubes were removed, covered with a plastic cap, and delivered to the chemistry department within 24 h for GC‐MS analysis.

Once collected in the ReCIVA® sorbent tubes, VOCs were processed by a thermal desorber (TurboMatrix™ 350; PerkinElmer, Waltham, Massachusetts, USA) fed with air (purity 99·988 per cent; SOL Group, Monza, Italy) and nitrogen (purity 99·999 per cent; Sapio Group, Taranto, Italy). Products were connected directly to a heated transfer line and then to GC‐MS (TRACE GC Ultra; Thermo Scientific, Waltham, Massachusetts, USA). Each tube was heated at 250°C for 20 min. Desorbed VOCs were then transferred at 200°C in splitless mode by the chromatograph injector using helium (purity 99·999 per cent; Sapio Group) as a carrier gas at a linear velocity of 0·6 cm/s. Separation and quantification of the desorbed VOCs was performed by coupling the chromatograph with a quadrupole mass spectrometer (ISQ™; Thermo Scientific) using a capillary column of 30 m × 0·25‐mm internal diameter with a 1·4‐μm film thickness (DB‐624 UI; Agilent Technologies, Santa Clara, California, USA), and at 40°C oven temperature for 5 min. The protocol is then ramped up to 160°C by 6°C per min (with 10 min at 160°C) and then increased by 6°C per min until it reaches 200°C. This heating is maintained for a further 15 min before being increased at 6°C per min to 220°C for a further 5 min. Temperatures reached at the transfer line and the quadrupole ion source were 280°C and 220°C respectively. Mass spectrometry was performed at 70 eV electron impact ionization energy in full‐scan mode, with a scan range of 40–250 atomic mass units. The Xcalibur™ software (Thermo Scientific) allowed acquisition and analysis of the data. In an effort to exclude extraneous contamination, on each sampling day three ReCIVA® steel tubes containing room air were sample‐tested before commencement of the breath sampling.

A preliminary study on the first 20 patients was performed to ascertain whether significant qualitative or quantitative differences in the chromatogram were present, desorbing and analysing each one of the four Tenax® tubes; no significant differences were found in the peaks among the four tubes in terms of intensity and resolution. However, to reproduce the same experimental conditions for each patient as far as possible, it was decided always to analyse the tube positioned in the same housing (position B) of the ReCIVA® unit. The other three tubes were also desorbed and analysed, and the chromatograms were saved and stored for control.

Individual VOCs were identified by injecting 1 μl of the working solution and calibrating as 50‐ or 100‐μg/ml aliquots with authenticated standards using the MS database of the National Institute of Standards and Technology. Stock solutions (20 mg/ml) of each volatile compound (purity at least 97 per cent; Sigma‐Aldrich, Milan, Italy) were prepared in methanol (purity at least 98 per cent; Sigma‐Aldrich), stored at 8°C, and diluted to prepare working solutions.

Targeted analysis was performed of putative colorectal cancer VOCs by depositing 50 ng of each analytical standard into a ReCIVA® sorbent tube. The tubes were analysed at random and in triplicate on the same day, as part of a reliability testing for all GC‐MS runs. Proposed ReCIVA® GC‐MS methods were tested by linear regression analysis using standardized solutions of suitable concentration, plotting the peak area against the concentration of analyte. After adding 1 ml of a standard solution containing the analytes at appropriate levels into the tubes, estimates were made of linearity, reproducibility, the limit of detection (LOD), the limit of quantification (LOQ), and threefold and tenfold basal signal‐to‐noise ratios.

### Statistical analysis

Quantitative variables approaching a normal distribution were reported as mean(s.d.) values. Comparison between patients with colorectal cancer and controls was done with Student's *t* test for independent samples. Sex and other categorical variables were reported as counts and percentages, with comparisons between independent groups performed with the χ^2^ test.

To assess the accuracy of the discriminatory capacity of VOCs for the detection of patients with colorectal cancer, the analysis was conducted in two stages. The first stage constructed a receiver operating characteristic (ROC) curve (with 95 per cent c.i., sensitivity and specificity) for each VOC and with the establishment of an individual cut‐off value for discrimination of colorectal cancer. The second stage involved cross‐validation using the ‘leave‐one‐out’ method, and applying logistic regression with the dependent variable being the status of the patients. The estimates were therefore classified as cancer *versus* no cancer, with each VOC acting as an independent variable in the analysis.

To evaluate the effect of multiple VOCs, multivariable logistic regression analysis was performed with stepwise selection of independent variables. Initially, all VOCs were dichotomized in accordance with their threshold, as had been determined in the univariable analysis. The model was then fitted according to the status of the patient (cancer *versus* no cancer) and to the dichotomized VOCs. This was then classified as a dummy variable where the number 1 was allocated to values predictive of cancer and the number 0 was applied for any remaining independent values, as well as according to age (more than 65 years *versus* 65 years or less). This system of analysis was collated as model A. A stepwise procedure was applied to select VOCs, with the criterion for entry into the model being a *P* value below 0·050.

Both *R* ^2^ and area under the ROC curve (AUC) values generated were used in evaluation of the model. Validation of the model was performed with the leave‐one‐out method, and the proportions of correctly classified cancer and no cancer cases were reported. Further modelling was employed to test reliability stages, fitting model B with the early cancer cases and model C with advanced stages, and by using the same independent variables as for model A. The ROC curve analysis for a single VOC was performed with MedCalc® version 19.1 (MedCalc Software, Ostend, Belgium), and the logistic regression and cross‐validation were conducted using SAS® 9.4 software (SAS Institute, Cary, North Carolina, USA). *P* < 0·050 was considered statistically significant.

## Results

Eighty‐nine patients with colorectal cancer and 90 non‐cancer controls were entered into the study between January 2017 and April 2019. Six patients with cancer were excluded because of a final histological diagnosis of adenoma. Thirty‐eight patients (46 per cent) had clinical cancer stages I–II and 42 (51 per cent) had stage III–IV disease; stage information was not available for three patients. The non‐cancer control patients had all undergone colonoscopy, a mean(s.d.) of 14(7) months before the start of the study. *Table* *1* shows the overall patient demographics and co‐morbidities. The groups were matched for sex, but the mean age of patients with colorectal cancer was significantly higher than that of the controls (*P* < 0·001). Hypertension was the only co‐morbidity that was more common in the patients with colorectal cancer than in the control group (*P* = 0·033).

**Table 1:** Demographics and co‐morbidities in colorectal cancer and control groups

	Colorectal cancer group (*n* = 83)	Non‐cancer control group (*n* = 90)	*P* †
**Age (years)** *	69·7(9·1)	58·7(13·4)	< 0·001‡
**Sex ratio (M** : **F)**	43 : 40	49 : 41	0·937
**Hypertension**	50 (60)	38 (42)	0·033
**Diabetes**	14 (17)	9 (10)	0·175
**Hypothyroidism**	8 (10)	4 (4)	0·168
**Cancer stage**			
I–II	38 (46)		
III–IV	42 (51)		
Missing	3 (4)		
**Smoker**	7 (8)	11 (12)	0·415

Values in parentheses are percentages unless indicated otherwise;

* values are mean(s.d.).

† χ^2^ test, except

‡ Student's *t* test.

GC‐MS analysis showed that 64 compounds were present in at least 50 per cent of colorectal cancer or non‐cancer breath samples collected from the ReCIVA® system and absent in compared room air samples. To identify VOCs able to discriminate between patients with cancer and those with no cancer, a non‐parametric Mann–Whitney *U* test was performed; this showed that levels of 38 of the compounds were significantly different in the two groups.

**Fig. 1:**
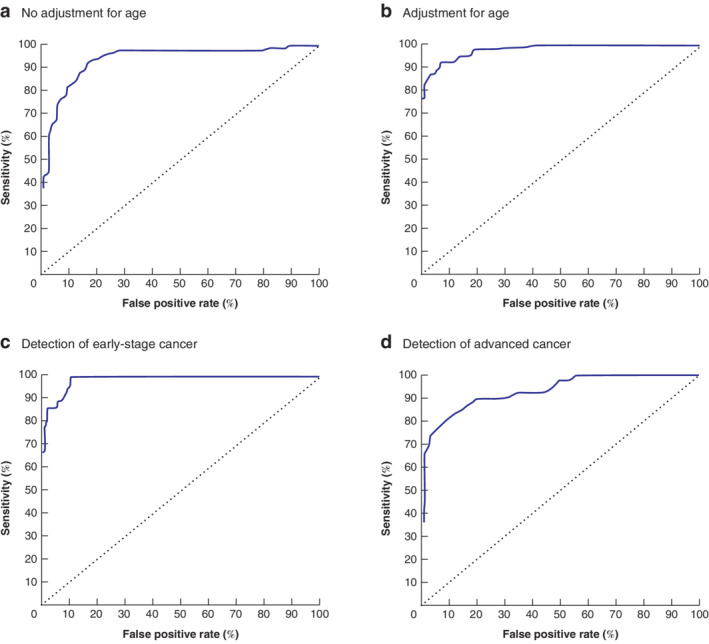
Receiver operating characteristic (ROC) curve analysis of the likelihood of discrimination for models A, B and C Multivariable models: **a** with no adjustment for age (likelihood = 151·03, *P* < 0·001, area under the ROC curve (AUC) = 0·963); **b** with adjustment for age class (model A) (likelihood = 173·45, *P* < 0·001, AUC = 0·979); **c** for detection of early‐stage cancer (model B) (likelihood = 113·63, *P* < 0·001, AUC = 0·985); **d** for detection of advanced cancer (model C) (likelihood = 88·51, *P* < 0·001, AUC = 0·933).

Using the relevant ROC curves for sensitivity and specificity and true predictive value with a cross‐validation univariable analysis, the VOCs were ordered from the most to the least accurate (by AUC and sensitivity for discriminating colorectal cancer from non‐cancer) (*Table* *2*). Fifteen VOCs were statistically significant in discriminating patients with colorectal cancer in univariable analysis. The highest AUC values were noted for tetradecane ethylbenzene and methylbenzene, octanal, nonanal and decanal. To evaluate the predictive value of the association of the diagnosis of colorectal cancer with particular VOCs, multivariable logistic regression analysis using discrete modelling was performed against age and colorectal cancer stage. *Table* *3* shows the analysis using the model A approach, comparing cancer and no cancer diagnosis. With this approach there was a significant likelihood of association (173·45, *P* < 0·001; *R* ^2^ = 0·642, AUC = 0·979) (*Fig*. *1b*). Cross‐validation of this model confirmed a 90 per cent rate of true positives, a 93 per cent rate of true negatives and a predictive value of 93 per cent. This selection procedure retained age group as well as the VOCs tetradecane, ethylbenzene, methylbenzene, acetic acid and 5,9‐undecadien‐2‐one, 6,10‐dimethyl (E), all of which were retained in the list of the top 14 VOCs from the univariable analysis.

**Table 2:** Volatile organic compounds identified in the exhaled breath of analysed patients

						Frequency (%)	
Peak no.	RT (min)*	Compound	Match (‰)	Probability (%)	Standard identity confirmation†	Colorectal cancer	No cancer
1	3·48(0·03)	Ethanol	919	87	Yes	30	64
2	8·58(0·03)	Acetic acid	959	69	Yes	100	83
3	10·88(0·04)	Methylbenzene	979	60	Yes	91	83
4	11·53(0·03)	Unidentified				100	100
5	11·60(0·02)	Dimethyl heptane	879	53		86	100
6	11·97(0·03)	Hexanal	989	65	Yes	31	58
7	12·54(0·03)	Octane, 4‐methyl	905	51		83	91
8	12·77(0·03)	Butanoic acid		75	Yes	60	62
9	13·04(0·04)	Ethylbenzene	954	71	Yes	78	82
10	13·22(0·03)	Xylene	908	61	Yes	51	58
11	13·85(0·03)	Unidentified				69	74
12	14·40(0·03)	2‐Butoxy ethanol	839	82		71	83
13	15·24(0·03)	Decane	913	54	Yes	52	50
14	15·74(0·02)	Benzaldehyde	930	92	Yes	77	88
15	16·07(0·02)	Octanal	888	60	Yes	71	84
16	16·31(0·02)	Undecane	922	57	Yes	58	55
17	16·42(0·03)	Unidentified				62	50
18	16·61(0·03)	2‐Ethyl‐1‐hexanol	850	63		77	70
19	16·99(0·03)	Unidentified				65	70
20	17·07(0·02)	3,3‐Dimethyl octane	907	55		74	91
21	17·91(0·04)	Nonanal	912	91	Yes	90	94
22	18·91(0·03)	Dodecane	934	55	Yes	91	100
23	20·00(0·02)	Decanal	879	51		97	91
24	20·58(0·02)	Benzoic acid	950	88	Yes	91	88
25	20·67(0·03)	4,6‐Dimethyl dodecane	907	53		74	66
26	21·01(0·03)	Tridecane	911	63	Yes	94	100
27	21·39(0·04)	Benzene, 1,3‐bis(1‐methylethenyl)	917	67		69	91
28	22·01(0·04)	Nonanoic acid	879	78	Yes	56	71
29	22·75(0·04)	Unidentified				66	65
30	23·16(0·03)	Tetradecane	934	53	Yes	83	90
31	23·24(0·03)	Unidentified				78	80
32	23·63(0·03)	2,4,4,6,6,8,8‐Heptamethyl‐1‐nonene	903	51		50	69
33	24·22(0·04)	Decanoic acid	867	77	Yes	53	59
34	24·40(0·02)	Ethanone, 1‐[4‐(1‐methyl‐ethenyl)‐phenyl]	815	61		71	74
35	24·65(0·03)	Pentadecane	921	53	Yes	60	70
36	25·52(0·03)	Nonadecane	907	51	Yes	51	59
37	25·77(0·02)	5,9‐Undecadien‐2‐one, 6,10‐dimethyl (E)	896	58		51	58
38	27·00(0·04)	Butyl hydroxy toluene	922	51		57	78

* Values are mean(s.d.).

† Authenticated using the National Institute of Standards and Technology library and standard injection.

RT, relative retention time.

**Table 3:** Multivariable logistic regression analysis of model A: colorectal cancer *versus* no cancer diagnosis

	Cut‐off value*	Regression coefficient (β)	s.e.	*P*
Age class (years)	> 65 *versus* ≤ 65	0·7294	0·1959	< 0·001
Tetradecane	≤ 0·39 *versus* > 0·39	0·6398	0·2025	0·002
Ethylbenzene	≤ 0·35 *versus* > 0·35	0·8162	0·2006	< 0·001
Methylbenzene	> 1·94 *versus* ≤ 1·94	0·593	0·1989	0·003
5,9‐Undecadien‐2‐one, 6,10‐dimethyl (E)	≤ 0·41 *versus* > 0·41	0·7661	0·2028	< 0·001
Benzaldehyde	≤ 4·06 *versus* > 4·06	0·6440	0·2114	0·002
Decane	≤ 0·77 *versus* > 0·77	0·5872	0·2060	0·004
Benzoic acid	> 0·94 *versus* ≤ 0·94	0·5423	0·2332	0·020
1,3‐Bis(1‐methylethenyl) benzene	> 0·52 *versus* ≤ 0·52	0·6035	0·2541	0·018
Decanal	≤ 5·99 *versus* > 5·99	−1·7920	0·6677	0·007
Unidentified compound	> 0·26 *versus* ≤ 0·26	1·9308	0·6931	0·005
2‐Ethyl‐1‐hexanol	> 2·51 *versus* ≤ 2·51	−0·9643	0·4826	0·046
Dodecane	> 3·51 *versus* ≤ 3·51	1·6296	0·5247	0·002
Ethanone, 1[4‐(1‐methylethenyl)phenyl]	≤ 0·39 *versus* > 0·39	1·3405	0·5494	0·015
Acetic acid	> 0·41 *versus* ≤ 0·41	3·0856	0·9442	0·001

* Cut‐off values for volatile organic compounds are expressed as peak area percentage, and listed in order of decreasing accuracy for colorectal cancer discrimination. Likelihood: 173·45, *P* < 0·001; area under the curve = 0·979 on receiver operating characteristic (ROC) and cross‐validation analysis.

*Table* *4* shows application of the multinomial model B; there was a significant likelihood of discrimination of early‐stage colorectal cancer compared with control samples (likelihood = 113·63, *P* < 0·001; *R* ^2^ = 0·603 per cent, AUC = 0·985) (*Fig*. *1c*). The VOCs entered into the model B format had already been selected from the model A analysis; they included tetradecane, ethylbenzene, methylbenzene, 5,9‐undecadien‐2‐one and tridecane. The model B analysis produced several further informative markers, including benzaldehyde, dodecane and butyl hydroxy toluene.

**Table 4:** Multivariable logistic regression analysis of model B: early‐stage colorectal cancer (36 patients) *versus* no cancer controls

	Cut‐off value*	Regression coefficient (β)	s.e.	*P*
Age class (years)	> 65 *versus* ≤ 65	1·7143	0·6776	< 0·001
Tetradecane	≤ 0·39 *versus* > 0·39	3·9856	1·1531	0·002
Ethylbenzene	≤ 0·35 *versus* > 0·35	3·2277	0·9774	< 0·001
Methylbenzene	> 1·94 *versus* ≤ 1·94	2·4972	0·8091	0·003
5,9‐Undecadien‐2‐one, 6,10‐dimethyl (E)	> 0·26 *versus* ≤ 0·26	2·9349	0·9292	< 0·001
Tridecane	≤ 0·74 *versus* > 0·74	−1·9505	0·8619	0·024
Benzaldehyde	≤ 0·77 *versus* > 0·77	1·9171	0·6965	0·006
Dodecane	> 3·51 *versus* ≤ 3·51	2·2698	0·8248	0·006
Butyl hydroxy toluene	≤ 13·82 *versus* > 13·82	−3·7177	1·1298	0·001

* Cut‐off values for volatile organic compounds are expressed as peak area percentage, and listed in order of decreasing accuracy for colorectal cancer discrimination. Likelihood: 113·63, *P* < 0·001.

*Table* *5* shows application of model C, comparing advanced colorectal cancer with no cancer. There was a significant likelihood of discrimination (likelihood = 88·51, *P* < 0·001; *R* ^2^ = 0·496 per cent, AUC = 0·933) (*Fig*. *1d*). Significant VOCs identified with the model C format included tetradecane, ethylbenzene, methylbenzene and benzaldehyde. Although benzoic acid did not reach significance in the univariable analysis in discriminating patients with colorectal cancer, it showed high specificity (81 per cent) in the model C assessment.

**Table 5:** Multivariable logistic regression analysis of model C: advanced colorectal cancer (442 patients) *versus* no cancer controls

	Cut‐off value*	Regression coefficient (β)	s.e.	*P*
Age class (years)	> 65 *versus* ≤ 65	1·2123	0·357	< 0·001
Tetradecane	≤ 0·39 *versus* > 0·39	1·1177	0·3275	< 0·001
Ethylbenzene	≤ 0·35 *versus* > 0·35	1·4618	0·3748	< 0·001
Methylbenzene	> 1·94 *versus* ≤ 1·94	1·3706	0·3673	< 0·001
Benzaldehyde	≤ 4·06 *versus* > 4·06	0·9318	0·3466	0·007
Benzoic acid	> 0·94 *versus* ≤ 0·94	1·1749	0·3940	0·003

* Cut‐off values for volatile organic compounds are expressed as peak area percentage, and listed in order of decreasing accuracy for colorectal cancer discrimination. Likelihood: 88·51, *P* < 0·001.

Cross‐validation of the three models used is shown in *Table* *6*. Model B resulted in the correct classification in 86 per cent of patients with early‐stage colorectal cancer and in 94 per cent of controls, with an overall positive predictive value (PPV) of 86 per cent. Model C showed a correct classification in 71 per cent of patients with advanced colorectal cancer and 97 per cent of controls, with an overall PPV of 91 per cent.

**Table 6:** Cross‐validation of colorectal cancer discrimination in the three models

	True condition	
	Colorectal cancer	No cancer	Total	True predictive value (%)	Sensitivity (%)	Specificity (%)
**Model A (all patients)**						
Cancer	74	6	80	93	90	93
No cancer	8	81	89			
Total	82	87				
**Model B (early‐stage cancer and no cancer)**						
Cancer	31	5	36	86	86	94
No cancer	5	82	87			
Total	36	87				
**Model C (advanced cancer and no cancer)**						
Cancer	30	3	33	91	71	97
No cancer	12	84	96			
Total	42	87				

In the analytical validation and threshold quantification of VOCs, ethylbenzene, methylbenzene and tetradecane were the only three compounds identified as statistically relevant in discriminating patients with colorectal cancer in all univariable and multivariable models. To quantify these three VOCs, linear regression analysis of the peak area *versus* analyte concentration was done. For this purpose, standard mixtures with concentration ranges that were dependent on the individual compound were added directly to virgin ReCIVA® sorbent tubes. *Table* *7* gives the analytical validation parameters of the GC‐MS method on standard reagents of the three compounds.

**Table 7:** Ethylbenzene, methylbenzene and tetradecane linearity and detection limits for proposed ReCIVA® gas chromatography–mass spectrometry method

						Relative standard deviation (%)	
Equation	*R* ^2^	Linear range (ng)	LOD (ng)	LOQ (ng)	Within‐day (*n* = 3)	Between‐day (*n* = 21)
Ethylbenzene	*Y* = 5·0*10^7^ × −5*10^8^	0·966	150–15000	50	163	4	17
Methylbenzene	*Y* = 4·0*10^7^ × +3*10^7^	0·994	15–1500	5	16	4	15
Tetradecane	*Y* = 6·0*10^7^ × −1*10^7^	0·997	10–1000	3·5	12	4	14

For limit of detection (LOD) the signal to noise ratio (S/N) was 3; for limit of quantification (LOQ) S/N was 10.

Threshold concentration values in the breath for the three selected substances (above or below which a patient could be affected by colorectal cancer with a 95 per cent c.i. of 90 per cent) were calculated as less than or equal to the LOQ value for ethylbenzene, greater than the LOD value for methylbenzene, and 53 ng or less for tetradecane.

## Discussion

Analysis of exhaled VOCs for the detection of early‐stage cancers would appear to be an attractive screening option because a non‐invasive approach such as a breath test is well tolerated. Despite an encouraging meta‐analysis^18^ reporting an overall high accuracy for exhaled VOCs in ancer diagnosis, there is currently no technical standardization of either breath collection or analysis. This study has identified specific exhaled VOCs as putative candidates in the discrimination of patients with colorectal cancer from controls. In addition, the reliability of the methodology appears to be independent of disease stage, highlighting its potential value as a diagnostic or screening tool.

Notably, a combination of age class (above 65 years) with a discrete pattern of 14 exhaled VOCs (tetradecane, ethylbenzene, methylbenzene, acetic acid, 5,9‐undecadien‐2‐one, 6,10‐dimethyl (E), decane, benzaldehyde, benzoic acid, 1,3 bis(1‐metiletenil) benzene, decanal, unidentified compound T22_75, dodecane, 2‐ethyl‐1‐hexanol and ethanone, 1[4‐(1‐methylethenyl)phenyl]) was able to discriminate patients with colorectal cancer from those without cancer with a high predictive value of 93 per cent overall. Further, this statistical model maintained a good predictive value even when the age of patients was not part of the analytical consideration (*Fig*. *1a*). Comparing the results of the univariable analysis with the three models obtained by multivariable logistic regression, three VOCs (ethylbenzene, methylbenzene and tetradecane) were found consistently to have a highly significant discriminatory ability in detecting patients with colorectal cancer.

In a previous study^4^ a customized breath sampler and a different GC‐MS column was employed and, in comparison with the present study, only a few VOCs with discriminatory capacity for colorectal cancer were identified. Differences between the findings of these studies are probably technical, as in the present study only the alveolar breath fraction was measured, with exclusion of extraneous contaminants. The use of Tedlar® bags (Sigma‐Aldrich) for breath collection in the earlier study^4^ may also have provided an opportunity for extraneous VOC contamination.

There are other potential reasons for the differences in these results that may be a consequence of the use of different capillary columns and oven profiles, as well as a different spectrometer. There has also been an important change in the statistical analysis used since the first report, from a probabilistic neural network to a ROC sensitivity curve. The present lack of standardization in breath collection, VOC measurement and statistical analysis all likely contribute to inconsistencies in the VOC profiles identified by different groups, and presently this limits the value of exhaled VOC patterns as a mass screening tool for diagnosis of colorectal cancer^18^. In this regard, another report^19^ identified a pattern of six VOCs capable of discriminating patients with colorectal cancer from those without cancer. In that report, breath samples were obtained via a simple mouthpiece containing a filter cartridge on the inspiratory port, collected into a 750‐ml polyvinyl fluoride sampling bag and then analysed by a GC‐MS (GC6890N; MS‐5975; Agilent Technologies), and the statistical analysis was performed using a principal components analysis (PCA) algorithm. Despite these important methodological differences, however, three of the VOCs identified in that study^19^ matched the present VOC pattern.

Another study^6^, published in 2014, compared the breath of 20 patients with colorectal cancer with that of 20 non‐cancer controls, and selected nine VOCs as discriminators for colorectal cancer, but only one VOC from that study, dodecane, was also isolated as a relevant VOC in the present study. A GC‐MS analytical platform was similarly used; however, their settings were different to the present ones and PCA was used for their statistical evaluation^6^. In 2016, Amal and colleagues^7^ identified another pattern of exhaled VOCs in a cohort of 65 patients with colorectal cancer and 122 controls; acetone and ethyl acetate were found in higher concentration, and ethanol and 4‐methyl octane in comparatively lower concentration, in the patients with cancer. Although the discriminatory value of ethanol in this study should be considered, there were methodological differences with the present analysis: the alveolar breath fraction was analysed using a two‐bed ORBOTM 420 Tenax® TA sorption tube and a GCMS‐QP2010 (Shimadzu, Kyoto, Japan) and an SLB®‐5 ms capillary column (Sigma‐Aldrich)^7^.

Another recent study^20^ used a different, sophisticated, analytical platform, selected ion flow tube mass spectrometry (SIFT‐MS), which permits real‐time measurement of trace concentrations of gases in humidified air samples. Even though there was no attempt to isolate the alveolar fraction of air, the authors specifically identified a single VOC, propanal, as a significant biomarker with high sensitivity (96 per cent) and specificity (76 per cent) for detection of colorectal cancer. That study^20^ documented a specific aldehyde molecule (one of the shorter volatile aldehydes and a structural isomer of acetone) that is difficult to detect with a standard GC‐MS platform. Propanal has also been implicated in the detection of other diseases, such as coeliac disease and bile acid diarrhoea^21^, ^22^.

The biological significance of the VOCs involved in carcinogenesis is extremely difficult to explain and would require further studies on the biochemical pathways behind their production. However, it seems clear that some of them, such as ethylbenzene and tetradecane, correlate significantly with the diagnosis of colorectal cancer, irrespective of stage; it may be speculated that their metabolic pathways are closely linked to the process of carcinogenesis, at least in the colon.

Although the search for a single specific colorectal cancer biomarker represents the ‘holy grail’ in VOC detection, and reflects a discretely measurable metabolic derangement within colorectal cancer cells^23^, cancer cells still use the same metabolic pathways as normal cells, although in quantitatively different ways. Hence, it may be more realistic to expect changes in the relative quantities of VOCs in colorectal cancer, rather than to isolate particular ‘cancer‐specific’ VOCs. Most studies concerning colorectal cancer detection have identified a patterned VOC signature, with VOCs such as nonanal and 1,3‐dimethylbenzene repeatedly detected as significant in a range of reports. It is also expected that discrepancies in the VOC profiles of patients with colorectal cancer could, at least in part, be the result of gastrointestinal dysbiosis, whereby complex interaction of the gut microbiome with the local inflammatory response is implicated in the transformation from normal to neoplastic tissue^24^. In a recently published study^25^, in which the VOCs exhaled by seven patients with colorectal cancer were compared with those produced from their own cancer tissue (the latter sampled *ex vivo* after surgery by headspace solid‐phase microextraction), concentrations of benzaldehyde and indole were significantly higher in cancerous tissue than in normal colonic mucosa, whereas ethylbenzene production was reduced in the cancer tissue. This finding suggests that normal colonic mucosa and cancer tissue can produce similar VOC patterns, even though their individual fingerprints may differ.

A limitation of this study is that patients and controls were recruited by a single centre, and came from the same geographical area with similar diet and environmental exposure; this could have a role in the pattern of exhaled VOCs. Therefore, a multicentre international trial on this topic is awaited. Currently, the equipment needed is expensive, and the data require complicated analysis, limiting the utility of this approach as a mass colorectal cancer screening tool. Some groups^16^, ^26^, ^27^ have reported the use of commercial or purpose‐designed electronic (e) noses to discriminate patients with colorectal cancer from those with advanced adenomas, as well as from non‐cancer controls. These results have, however, been mixed, as each chemical sensor used in an e‐nose can be activated by several VOCs belonging to the same chemical class of compound.

Confirmation of the pattern of VOCs identified in this paper in a multicentre international study may support the next step in the clinical application of breath sampling in colorectal cancer screening, which is to create a smart, inexpensive, online sensory device with reproducibly high discrimination for colorectal cancer. The binary result should be capable of directing a further screening investigation, such as colonoscopy.
